# Multi-modality image-based computational analysis of haemodynamics in aortic dissection

**DOI:** 10.1007/s10237-015-0729-2

**Published:** 2015-09-28

**Authors:** Desmond Dillon-Murphy, Alia Noorani, David Nordsletten, C. Alberto Figueroa

**Affiliations:** Department of Biomedical Engineering, King’s College London, London, SE1 7EH UK; Departments of Surgery and Biomedical Engineering, University of Michigan, North Campus Research Complex B20-211W, Ann Arbor, MI 48109 USA

**Keywords:** CFD, Aortic dissection, Multi-scale modelling, Cardiac work load, Intimal tears

## Abstract

Aortic dissection is a disease whereby an injury in the wall of the aorta leads to the creation of a true lumen and a false lumen separated by an intimal flap which may contain multiple communicating tears between the lumina. It has a high associated morbidity and mortality, but at present, the timing of surgical intervention for stable type B dissections remains an area of debate. Detailed knowledge of haemodynamics may yield greater insight into the long-term outcomes for dissection patients by providing a greater understanding of pressures, wall shear stress and velocities in and around the dissection. In this paper, we aim to gather further insight into the complex haemodynamics in aortic dissection using medical imaging and computational fluid dynamics modelling. Towards this end, several computer models of the aorta of a patient presenting with an acute Stanford type B dissection were created whereby morphometric parameters related to the dissection septum were altered, such as removal of the septum, and the variation of the number of connecting tears between the lumina. Patient-specific flow data acquired using 2D PC-MRI in the ascending aorta were used to set the inflow boundary condition. Coupled zero-dimensional (Windkessel) models representing the distal vasculature were used to define the outlet boundary conditions and tuned to match 2D PC-MRI flow data acquired in the descending aorta. Haemodynamics in the dissected aorta were compared to those in an equivalent ‘healthy aorta’, created by virtually removing the intimal flap (septum). Local regions of increased velocity, pressure, wall shear stress and alterations in flow distribution were noted, particularly in the narrow true lumen and around the primary entry tear. The computed flow patterns compared favourably with those obtained using 4D PC-MRI. A lumped-parameter heart model was subsequently used to show that in this case there was an estimated 14 % increase in left ventricular stroke work with the onset of dissection. Finally, the effect of secondary connecting tears (i.e. those excluding the primary entry and exit tears) was also studied, revealing significant haemodynamic changes when no secondary tears are included in the model, particularly in the true lumen where increases in flow over $+200\,\%$ and drops in peak pressure of 18 % were observed.

## Introduction

In aortic dissection, a tear in the aortic intima creates a true and false lumen for blood flow. Regardless of the type of dissection (Stanford type A or B), patients have a poor long-term prognosis with 50 % mortality at 5 years (Tsai et al. 2007). The majority of deaths occur due to false lumen expansion, further dissection and rupture. Current anatomical predictors of adverse outcomes include initial aortic size, a patent (or partially thrombosed false lumen) and Marfan’s syndrome. Such anatomical predictors of poor outcomes are used to customise follow-up strategies and optimise treatment planning. However, physiological indices such as intra-aortic haemodynamics (pressure and flow) may, in fact, provide improved patient-specific predictors of outcomes.

A number of studies (Karmonik et al. 2008; Rudenick et al. 2010; Karmonik et al. 2010; Cheng et al. 2010; Karmonik et al. 2011b; Tse et al. 2011; Karmonik et al. 2011a, 2012; Chen et al. 2013; Karmonik et al. 2013; Alimohammadi et al. 2014) have shown that analysis of blood flow through patient-specific geometries using computational fluid dynamics can yield significant insight into the complex haemodynamics present in dissection patients which may be difficult or impossible to quantify with other means. For example, in a longitudinal study involving a dissection patient with image data acquired pre- and post-aneurysmal development Tse et al. (2011) investigated the influence that the aneurysm has on haemodynamics. A similar study can be found in Karmonik et al. (2012). Karmonik also investigated how flow and pressure patterns in the vicinity of the dissection are affected when either the entry tear or exit tear is occluded (Karmonik et al. 2010), haemodynamic changes pre- and post-EVAR (Karmonik et al. 2011b), as well as a comparison between haemodynamics in a healthy subject and those in a patient with dissection (Karmonik et al. 2013). Rudenick et al. (2013) performed a combined experimental and computational study using an idealised rig of a dissection, comparing measured and simulated flows in both the true and false lumina, showing good agreement. In Chen et al. (2013), the haemodynamics of a dissection patient were studied in a model that included primary entry and exit tears, as well as a secondary tear. Lastly, Alimohammadi et al. (2014) performed a study in a simpler dissection model using coupled RCR Windkessel models at the outlet branches.

Secondary intimal tears in the dissection flap are thought to occur due to avulsion around branching vessels as the dissection propagates (Willoteaux et al. 2004). Studies have found that patients with dissection generally present with more than two tears (Quint et al. 2003; Khoynezhad et al. 2010). However, as it has been shown, tears may be difficult to identify in the medical image data, due to the small size and potential motion artefacts in the septum. In this study, we used a combination of two imaging modalities, CTA and 4D PC-MRI, to try to detect the location of these secondary tears.

In this paper, the haemodynamic impact of different aortic dissection morphometric parameters is investigated by conducting the following three novel studies:

1. An analysis of the haemodynamic changes introduced by the dissection septum in the descending aorta. Here, a comparison of haemodynamic predictions was made between a multi-branched, multi-scale model of aortic dissection and a baseline ‘healthy’ aortic model created by virtually removing the septum. This approach enables us to investigate specific changes in blood flow, pressure, wall shear stress, etc. directly attributable to the dissection.
2. An estimation of the additional stroke work imposed by the aortic dissection on the heart using a tuned lumped-parameter heart model.
3. A quantification of the impact of the often ignored secondary tears on aortic haemodynamics. These tears, typically found in the vicinity of visceral branches, are difficult to visualise due to limitations in the image resolution and motion artefacts. In this study, three different dissection models with varying number of tears were investigated. The results demonstrate that while these tears may be difficult to image, they can, if present, have a significant influence on the haemodynamics.

In all previous studies on aortic haemodynamics, a validation of numerical predictions against in vivo imaging data is lacking. We aim to address this and validate our results by using rich medical imaging data (computed tomography, 2D PC-MRI and 4D MRI) to assess the quality of our computer simulations, comparing simulated and measured flows in several true lumen and false lumen locations in the descending aorta of a patient with an acute type B dissection.

**Fig. 1:**
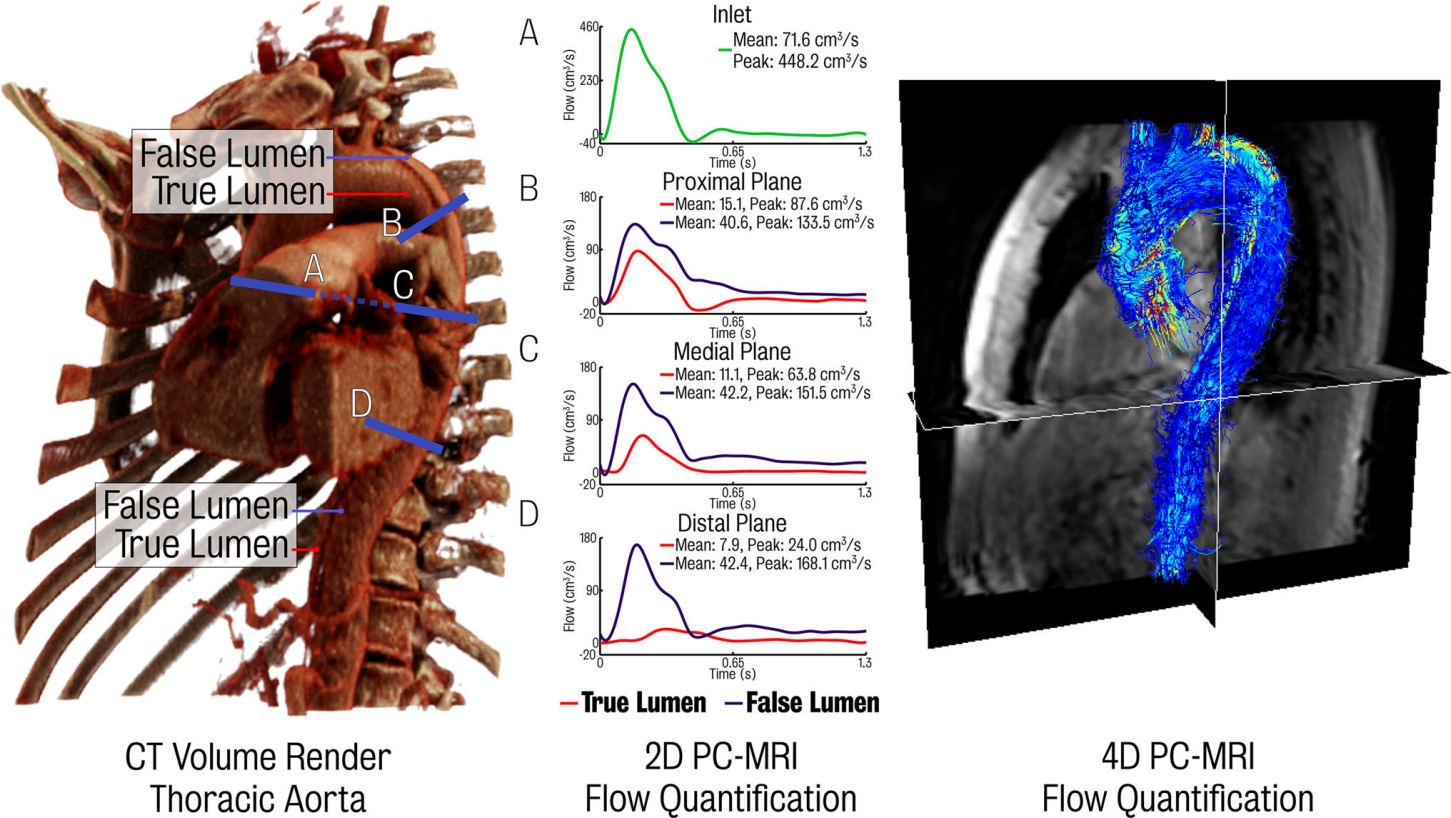
*Left* CT volume render of the thoracic aorta illustrating the dissection septum. *Centre* the 2D PC-MRI data at aortic inflow and several locations down the aorta. *Right* full aorta 4D PC-MRI data

The structure of this paper is as follows: in Sect. 2, details on patient and imaging data, geometric modelling approach and computational tools are provided. In Sect. 3, the above-mentioned studies are presented. Lastly, the paper is concluded by discussing a set of limitations on both the imaging and the modelling sides that must be overcome to enhance the accuracy of image-based modelling of aortic dissection.

## Material and methods

### Patients details

A 49-year-old male subject presented with an acute type B dissection originating 5 mm distal of the left subclavian artery, extending the full length of the descending aorta and terminating 5 mm distal to the left iliac bifurcation. The long axis of the dissection septum progressively rotates approximately $90^{\circ }$ clockwise along the length of the descending thoracic aorta, bisecting it largely in the sagittal direction in the abdominal region. A number of vessels branch from the false lumen (left renal artery, inferior mesenteric artery and medial sacral artery), while the remaining branch from the true lumen. It should also be noted that several vessels in the coeliac trunk region (coeliac trunk, superior mesenteric artery) have, due to their proximity of the dissection flap, a significantly “pinched” appearance at the branching location from the aorta, resulting in an oval cross section with the short axis aligned with the transverse plane.

The patient was previously prescribed antihypertensive drugs (Amlodipine 10 mg, Candesartan 4 mg) to treat a history of high blood pressure pre the dissection event. On presentation, the patient was administered additional intravenous antihypertensive medication to acutely control blood pressure.

**Fig. 2:**
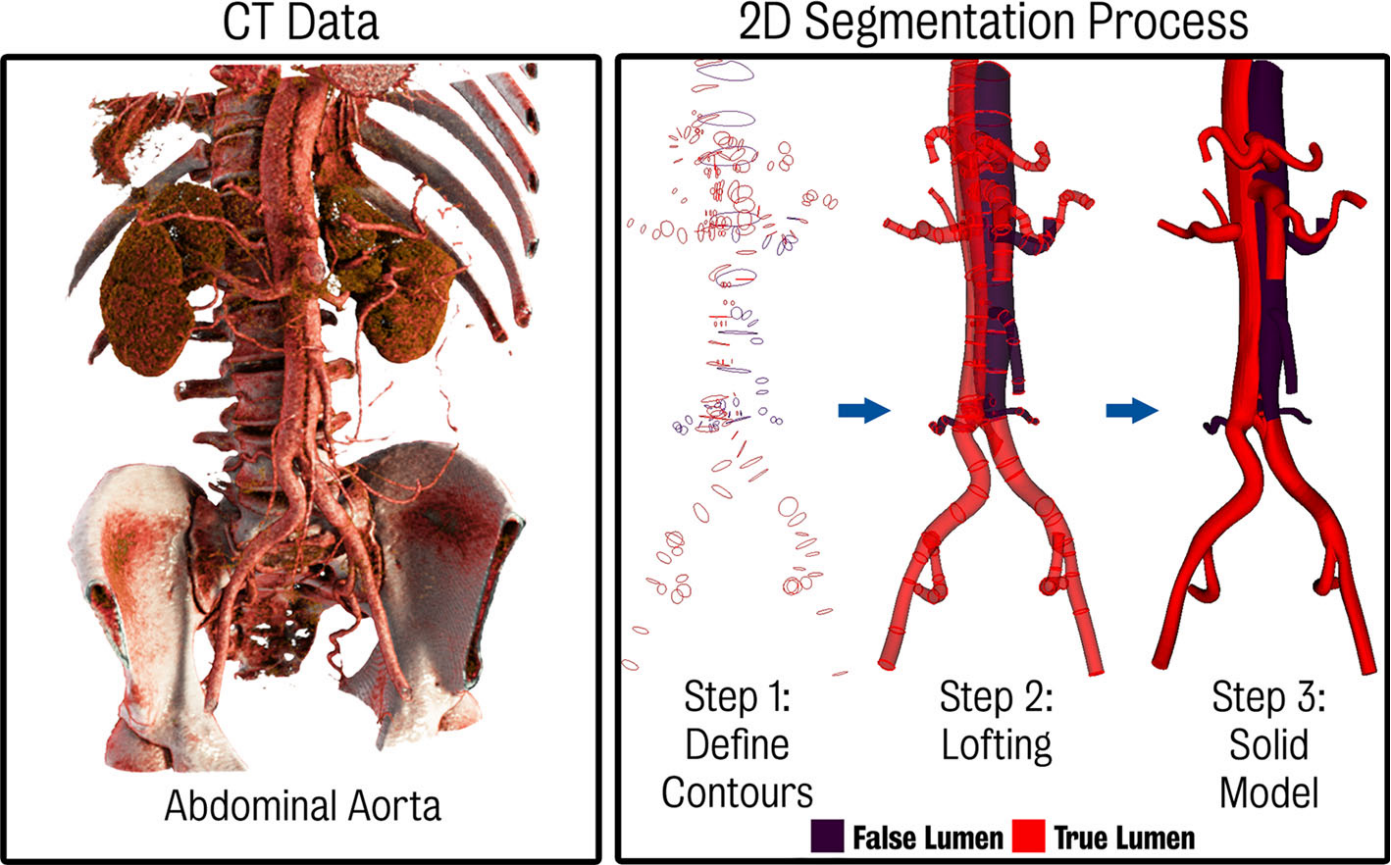
*Left* CTA showing the anatomy of the dissection in the abdominal aorta. The small size and motion of the septum make it difficult to clearly delineate the true and false lumen. *Right* 3D solid model creation process using a 2D segmentation approach. In step 1, contours of the lumina are created. These contours are then lofted together (step 2) to create a non-uniform rational B-spline (NURBS) analytical representation of each vessel. Finally, (step 3) the lofted surfaces are joined and blending operations using fixed radius fillets are employed to remove sharp edges between branching vessels. The false lumen and vessels perfused from the false lumen are coloured in *purple*, while all other vessels are coloured in *red*

### Imaging data

#### Anatomy: CT data

A 40-row multi-slice scanner (Brilliance 40; Philips Medical Systems, Cleveland, Ohio) was used to image the full aorta in two separate scans acquired using first-pass bolus-tracked contrast-enhanced images during one breath hold: one capturing the thoracic aorta and the second the abdominal aorta (see Figs. 1, 2); 100 mL of iohexol contrast agent (Omnipaque 350; GE Healthcare, Oslo, Norway) was delivered at 4 mL/s using a power injector (Medrad Spectris, Siemens Medical Solutions, Malvern, Pa). The scan was automatically triggered when the contrast enhancement reached 150 HU. Settings for both the thoracic and abdominal scans are listed in Table 1.

**Table 1:** Scan settings for both thoracic and abdominal CT scans of an aortic dissection subject

Property	Thoracic	Abdominal
FOV	376 mm	280 mm
In plane resolution	$512\times 512$	$512 \times 512$
Slice thickness	2 mm	0.9 mm
Tilt	$0^{\circ }$	$0^{\circ }$
Tube voltage (KPV)	120 kV	120 kV
Tube current	412 mA	221 mA
Acquisition	Cephalocaudal	Cephalocaudal

#### Flow data: MRI data

In this work, 2D PC-MRI data were used to quantify blood flow at four different locations, while 4D PC-MRI data were used to perform a qualitative comparison between imaging and CFD-derived velocity fields. All image data were collected using the methods presented in Clough et al. (2012) and are summarised here for completeness.

A thirty-two element coil Achieva 3T scanner (Philips Healthcare, Best, the Netherlands) was used to acquire the images. Image reconstruction was performed using a Philips scanner console and commercially available software. Eddy currents and Maxwell phase offsets in the 2D PC-MRI and 4D PC-MRI data were corrected for using standard Philips software algorithms (Bernstein et al. 1998; Markl et al. 2003; Gatehouse et al. 2010).

Flow-encoded through-pane images were acquired at three positions perpendicular to the longitudinal axis of the aorta capturing flow. These locations included the ascending aorta just distal to the aortic valve (this plane also acquired flow in the descending aorta at the level of the T-4 vertebra), the descending aorta, 15 mm distal to the left subclavian and the descending aorta at the level of the T-7 vertebra. The position of these planes is noted in blue in Fig 1. ECG-gated data were later allotted to 25 cardiac phases (details given in Table 2).

**Table 2:** 2D PC-MRI scan properties

Property	Value
Field of View (FOV)	$350 \times 350\,\hbox {mm}^{2}$
Voxel size	$1.74 \times 1.99\,\hbox {mm}^{2}$
Slice thickness	10 mm
Velocity encoding	150 cm/s
TR/TE	5.0/3.0 ms
Temporal resolution	52 ms
Number of signal averages	2

The reconstructed images were checked for phase wraps immediately after acquisition. If present, velocity encoding was increased to ensure that encoding for the peak velocity was attained. Stroke volume and heart rate were found to be 94 mL and heart rate 47 BPM, respectively. This corresponds to a cardiac output of 4.4 L/min. Velocity data were processed using the multi-dimensional MRI phase-contrast flow visualisation program, GTFlow (GyroTools LLC).

The entire thoracic aorta was covered using an oblique sagittal slab 4D MRI anatomy scan (see Fig. 1). ECG-gating and respiratory navigator were used for data acquisition of a single respiratory phase (5-mm gating window at end expiration). ECG-gated data were later allotted to 25 cardiac phases. A flow-sensitive, gradient and radio frequency-spoiled gradient echo (phase-contrast) sequence was used (details given in Table 3).

**Table 3:** 4D PC-MRI scan properties

Property	Value
Field of view (FOV)	$320 \times 320\times 85\,\hbox {mm}^{3}$
Voxel size	$2.0 \times 2.04\times 8\,\hbox {mm}^{3}$
Flip angle	$45^{\circ }$
Velocity encoding	150 cm/s
TR/TE	3.0/2.0 ms
Temporal resolution	52 ms

### Geometric modelling

In order to create a geometric CAD model, the 2D segmentation paradigm originally introduced in Wang et al. (1999) was utilised. Using this approach, paths were defined through roughly the centreline of the vessels to be included in the model. Then, a 2D automatic segmentation operation along the paths was performed at a number of discrete locations. This 2D segmentation step provides the contours of true and false lumina of the dissection. Lastly, lofted surfaces are obtained using non-uniform rational B-splines (NURBS) to produce an analytical and smooth solid model that must then be meshed. The right panel of Fig. 2 depicts the various steps of this segmentation approach. The false lumen and vessels perfused from the false lumen are coloured in purple, while all other vessels are coloured in red.

**Fig. 3:**
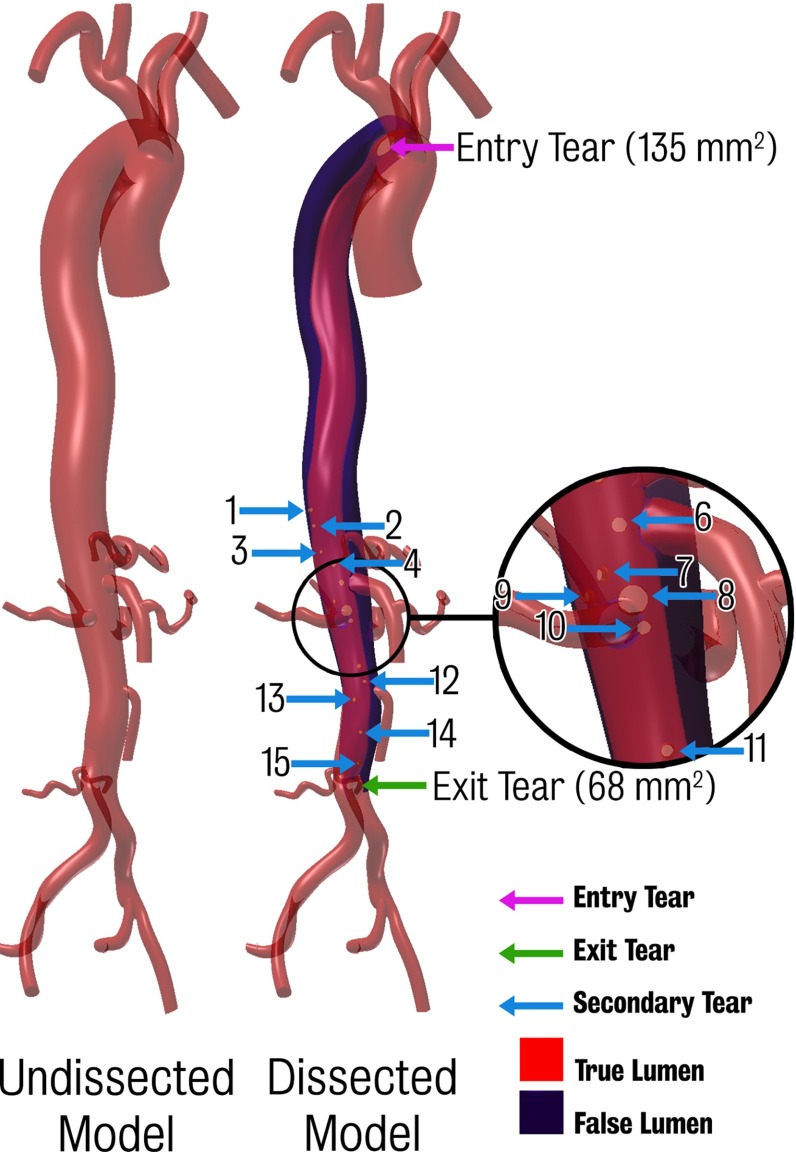
Undissected and dissected models. *Highlighted* are the entry tear, exit tear and 15 secondary tears

Several models were created using this methodology. A baseline “dissected model” containing the primary entry and exit tears as well as 15 secondary connecting tears were generated from the CT data. Most of these secondary tears were located in the visceral region. These tears generally form in regions where the septum separates from the wall in the vicinity of a branching vessel, and are believed to be the remnants of the original entry to the branching vessel (Willoteaux et al. 2004). A large number of connecting tears may be observed near the coeliac trunk, renal artery, etc. (Tam et al. 1998; McMahon and Squirrell 2010; Takami et al. 2012). The size of the main entry and exit tears was 135 and $68\,\hbox {mm}^{2}$, respectively. This corresponds to effective diameters of 13 and 9.3 mm, respectively. From this baseline “dissected model”, an “undissected model” was created by virtually removing the septum from the dissected model and keeping the outer boundary of its contours, thereby assuming that there are no significant changes in diameter between dissected and undissected models. Both dissected and undissected models have dimensions within the normal anatomical range. The “undissected model” is therefore representative of a healthy aorta and will be used to investigate the acute haemodynamic changes introduced by the dissection. Both these models are shown in Fig. 3. Furthermore, to address the difficulty in detecting connecting tears between the lumina, and to investigate their potential impact on the haemodynamics, two additional dissected models featuring different numbers of secondary connecting tears were also created. These models will be discussed in more detail in Sect. 3.

**Fig. 4:**
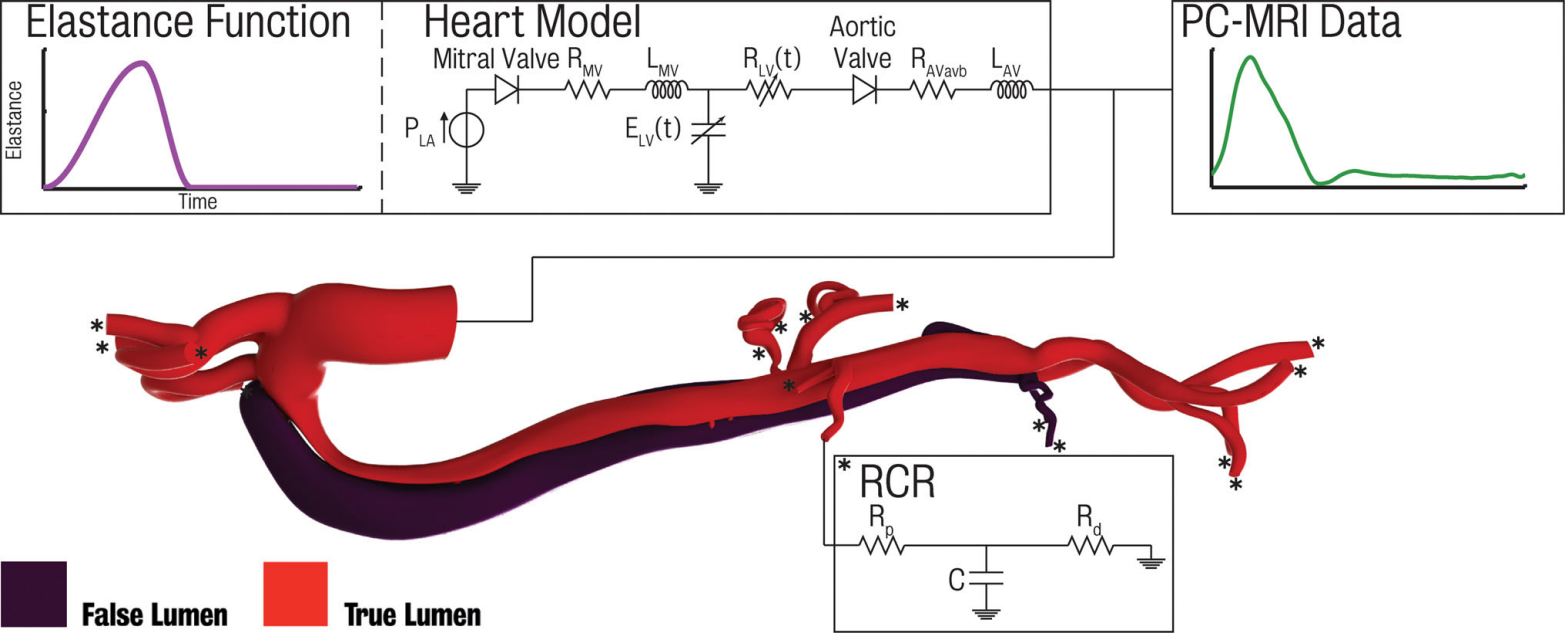
Schematic showing inlet and outlet boundary conditions used in this paper. A three-element Windkessel lumped-parameter model was specified on each outlet face of the arterial model. Inflow conditions were prescribed from either 2D PC-MRI data or via a lumped-parameter heart model. Tuning the elastance function the heart model allows for a close match with the measured 2D PC-MRI flow data

The models created in this study are arguably the most complex and geometrically sophisticated aortic models to date. Previous studies of dissection did not include the branching vessels around the coeliac trunk region (Karmonik et al. 2008, 2011a; Alimohammadi et al. 2014). It has however been noted that in the vicinity of branches there exists highly complex flow fields which may have a significant influence on perfusion (Shahcheraghi et al. 2002; Tse et al. 2011). Therefore, the models presented here were constructed to preserve as much of the branching vasculature thought necessary for accurate modelling, resulting in 17 outlets in total, including upper branch vessels (left and right subclavian and carotid arteries), visceral vessels (coeliac trunk, superior mesenteric, inferior mesenteric, left and right renal arteries) and iliac vessels (left and right internal and external iliac).

### Computational fluid dynamics

#### Coupled multi-domain method for blood flow simulation

In this section, we describe the coupled multi-domain method for blood flow simulation used in this paper. This method uses a stabilised finite element formulation to solve the incompressible Navier–Stokes equations in a 3D rigid domain reconstructed from image data, coupled to simpler, reduced-order models of the circulation at the inlet and outlet faces of the computational domain. It therefore represents a multi-scale (or multi-resolution) approach to blood flow modelling, as flow and pressure are calculated over the entire systemic circulation. Details of this method can be found elsewhere (Vignon-Clementel et al. 2006). For the sake of conciseness, we present the strong form as

$$\begin{aligned}&\rho \dot{\varvec{v}}+\rho {\varvec{v}}\cdot \nabla {\varvec{v}}=-\nabla p+\nabla \cdot \varvec{\tau } \\&\nabla \cdot {\varvec{v}}=0 \end{aligned}$$

where $\rho$ is the blood density (set at 1.060 kg/$\hbox {m}^{3}$ for each simulation), ${\varvec{v}}$ and *p* and represent the blood velocity and pressure at time *t* and $\varvec{\tau } =\mu \left( {\nabla {\varvec{v}}+\left( {\nabla {\varvec{v}}} \right) ^{\mathrm{T}}} \right)$ is the viscous stress tensor of a Newtonian fluid, where $\varvec{\mu }$ is the blood viscosity (set to $4\times 10^{-3}\hbox {Pa}\,\hbox {s}$). Body forces are set to zero. With appropriate choices for boundary conditions, and for the solution and test functional spaces *S*, *W* and *P*,  the strong form yields the following weak form:

$$\begin{aligned}&\mathop \int \limits _\Omega {\left\{ {\varvec{w}}\cdot \left( {\rho \dot{\varvec{v}}+\rho {\varvec{v}}\cdot \nabla {\varvec{v}}} \right) +\nabla {\varvec{w}}:\left( {-p{\varvec{I}}+\varvec{\tau }} \right) -\nabla q\cdot {\varvec{v}} \right\} \hbox {d}V}\nonumber \\&\quad +\mathop \int \limits _{\Gamma _g } {q{\varvec{v}}\cdot {\varvec{n}}\hbox {d}A} -\mathop \int \limits _{\Gamma _h } {{\varvec{w}}\cdot {\varvec{t}}^{h}\hbox {d}A} +\mathop \int \limits _{\Gamma _h } {q{\varvec{v}}\cdot {\varvec{n}}\hbox {d}A+\hbox {Stab}=0} \nonumber \\&\quad \forall {\varvec{x}}\in \Omega ,\forall {\varvec{t}}\in \left[ {0,T} \right] \end{aligned}$$

where ${\varvec{w}}\in W$ and $q\in P$ are test functions for the momentum and mass conservation equations, respectively. $\varOmega$ represents the 3D domain generated from the CT data, respectively. $\varGamma _g$ is a Dirichlet boundary (typically the inflow) where the test function ${\varvec{w}}$ vanishes and $\varGamma _h$ is a Neumann boundary where a traction

$$\begin{aligned} {\varvec{t}}^{h}=\left( {-p{\varvec{I}}+\varvec{\tau } } \right) \cdot {\varvec{n}}={\varvec{h}}\left( {{\varvec{v}},p,{\varvec{x}},t} \right) \end{aligned}$$

is prescribed via the 3D–0D domain interface coupling conditions. Figure 4 shows a schematic of the boundary conditions utilised in this paper. The term “Stab” refers to the stabilisation terms of the SUPG formulation (Whiting and Jansen 2001) utilised in our in-house software CRIMSON (www.crimson.software 2015).

A three-element Windkessel model was coupled to the outlet face of each of the branches. In this model, the distal vasculature (small arteries and arterioles) is represented via a zero-dimensional electric circuit analogue that uses resistors to represent haemodynamic resistance and capacitors to capture the vessel compliance (Formaggia et al. 1999; Westerhof et al. 2009; Vignon-Clementel et al. 2010). This simple model is one of the most commonly used approaches for outflow boundary condition specification in the simulation of haemodynamics (Stergiopulos et al. 1992; Alastruey et al. 2007; Xiao et al. 2013). Representation of realistic flow and pressure waveforms is achieved via proper specification of the model parameters. The three-element Windkessel model relates flow *Q* and mean pressure *P* over a cross section via the following operator:

$$\begin{aligned} \left[ \begin{array}{c} {P_d -P_c } \\ {C\frac{\hbox {d}P_\mathrm{d} }{\hbox {d}t}} \end{array} \right] =\left[ \begin{array}{c} {R_p Q} \\ {Q-\frac{P_\mathrm{c} -P_\mathrm{d} }{R_\mathrm{d} }} \end{array} \right] \end{aligned}$$

where *C* is the compliance of the distal vasculature, $R_\mathrm{p}$ and $R_\mathrm{d}$ are the proximal and distal resistances, respectively, $P_\mathrm{d}$ is a distal reference pressure (ground value in the Windkessel model), $P_\mathrm{C}$ is the Windkessel mid-pressure and *Q* is the flow going through the vessel. Stabilisation at the 3D–0D interface is provided by a flux term on outflow/inflow boundary of the 3D domain on an element-by-element basis as described in Hughes and Wells (2005).

**Fig. 5:**
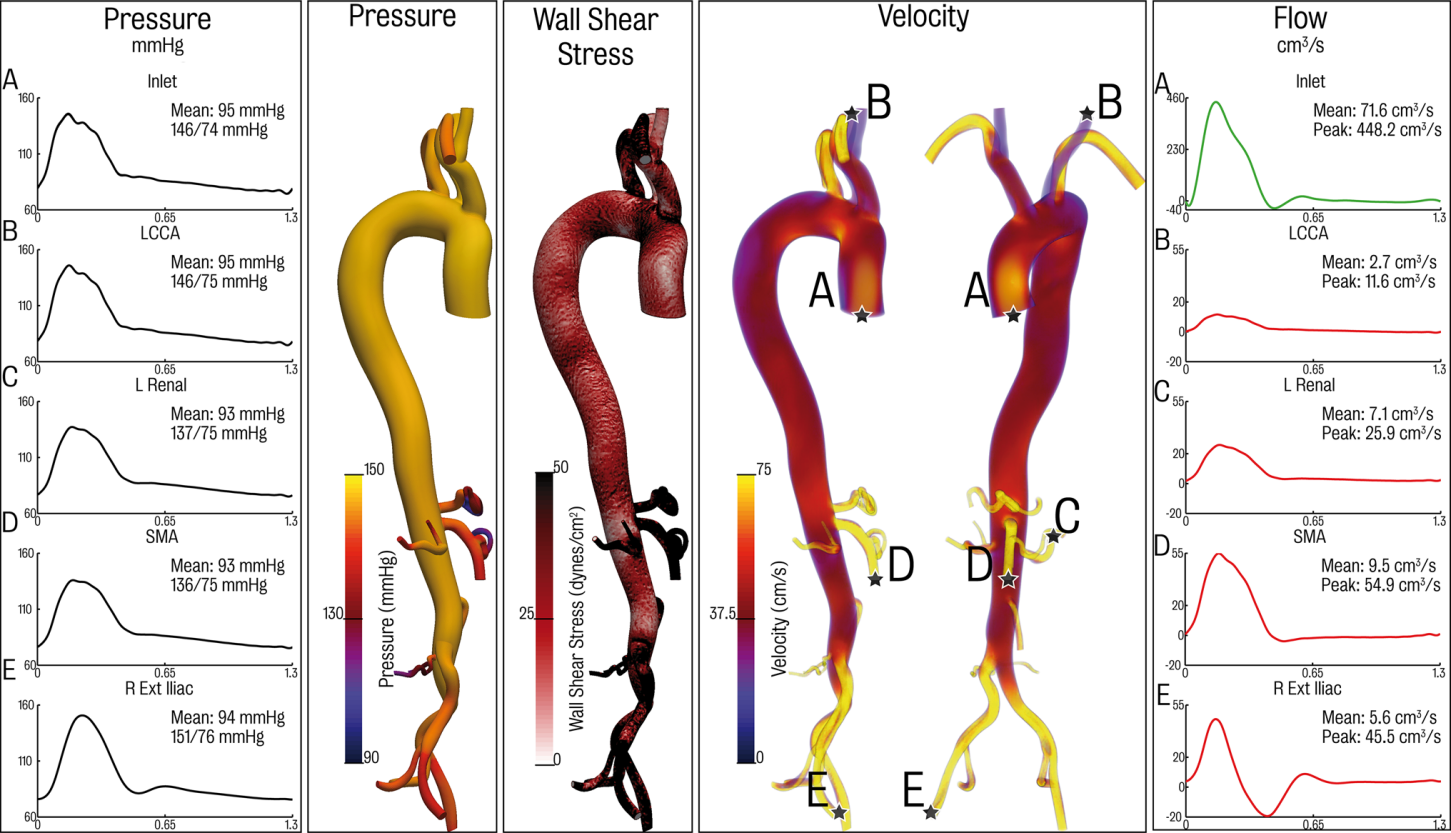
Visualisations of pressure, wall shear stress and velocity at peak systole for the baseline undissected model. Shown also are pressure and flow waveform plots at the inlet, left common carotid, left renal, superior mesenteric artery and right external iliac

Regarding the inflow conditions, two different approaches were adopted:

1. An inflow waveform was prescribed at the inlet face of the model. The waveform was derived from the 2D PC-MRI data measured just distally to the aortic valve and was mapped to a time-varying parabolic velocity profile.
2. A lumped-parameter model of the left heart tuned to match the flow waveform reconstructed from PC-MRI.

A number of reduced-order heart models have been previously proposed (Hunter et al. 2003; Ottesen et al. 2004; Formaggia et al. 2006; Kerckhoffs et al. 2006; Kim et al. 2009). The one presented in this paper is an implementation of that developed by Lau and Figueroa (2015). This model consists of a constant pressure source ($P_{\mathrm{LV}}$) representing the left atrium, a time-varying capacitor representing the left ventricular elastance ($E_{\mathrm{LV} }(t)$) and diodes representing the mitral and aortic valves. The pressure in the left ventricle was calculated as a function of the elastance $E_{\mathrm{LV}}$, left ventricular volume $V_{\mathrm{LV}}$ and the unstressed left ventricular volume $V_{\mathrm{u},\mathrm{LV}}$,

$$\begin{aligned} P_{\mathrm{LV}} (t)=E_{\mathrm{LV}} (t)\left( {V_{\mathrm{LV}} (t)-V_{\mathrm{u},\mathrm{LV}} } \right) \end{aligned}$$

Our model for the ventricular elastance $E_{\mathrm{LV} }(t)$ is based upon trigonometric functions, as detailed in Pope et al. (2009). The model also includes a time-varying resistance $R_{\mathrm{LV}}$ that accounts for viscous pressure losses. This resistance is proportional to a constant $k_{\mathrm{LV}}$, the left ventricular pressure $P_{\mathrm{LV}}$ and the left ventricular flow $Q_{\mathrm{LV}}$:

$$\begin{aligned} R_{\mathrm{LV}} (t)=k_{\mathrm{LV}} P_{\mathrm{LV}} Q_{\mathrm{LV}} \end{aligned}$$

The heart model was implemented in the context of the variational multi-scale approach of the multi-domain method (Vignon-Clementel et al. 2006) where 0D models are implicitly incorporated into the weak form of the problem. This leads to a monolithic implementation where the boundary integrals containing the reduced-order models contribute to the left hand-side matrix, and iterations continue until adequate convergence for the coupled problem is achieved (typically 4–6 nonlinear iterations per time step). By tuning the parameters of the heart model, specifically the time to maximum elastance and the maximum elastance value, it is possible to adjust the resulting inflow wave form to produce the desired levels of peak and mean flow. Thus, this approach enables us to reproduce a measured flow waveform without imposing it directly. In doing so, and by using standard values for the heart model published in the literature, we have a method that enables the estimation of cardiac workload, which will make it possible to estimate alterations in cardiac stroke work for this patient when changes in afterload resulting from the sudden onset of the dissection morphology occur (Lau and Figueroa 2015).

#### Mesh generation and time integration strategy

In blood flow modelling, velocity patterns, wall shear stress and pressure gradients can be greatly affected by coarse meshes that ultimately render the blood unphysical numerical viscosity (Taylor and Figueroa 2009). In this work, a two-step strategy was used for the finite element mesh generation. An initial mesh was created for each model using boundary layer and local curvature-based refinement. Simulations were run using the 2D PC-MRI flow data as inflow boundary conditions, and followed by a field-driven mesh adaptation technique to refine the mesh in areas of large velocity gradients (Sahni et al. 2006). The final unstructured field-adapted meshes for each model ranged between 2.6 million tetrahedral elements and 0.4 million nodes in the “undissected model” to 13.2 million tetrahedral elements and 2.4 million nodes in the “dissected model”.

The generalised-$\alpha$ method time integrator was used to advance the solution in time (Jansen et al. 2000). Analyses were run using between 2600 and 10,400 time steps per cycle (time step size between 500 $\upmu$s and 125 $\upmu$s for a cardiac cycle of 1.3 s) on 256 cores of a 640 core SGI Altix-UV HPC with Nehalem-EX architecture. Residual control, which limits the sum total of all the individual nodal residuals for the entire mesh, was set to $1\times 10^{-3}$. Each cardiac cycle took approximately between 4 and 18 h to run. For each case, simulations were run between 3 and 7 cardiac cycles until a cycle-to-cycle periodic solution in flow and pressures was achieved.

## Results

### Study 1: Haemodynamic alterations in aortic dissection

#### Baseline haemodynamics in undissected aorta

The simulation considered first was that of the undissected aorta model created from the aortic dissection image data by virtually removing the septum while keeping all aortic contours unchanged. As no patient-specific data were available for the undissected state, the same flow data derived from the dissected 2D PC-MRI study were applied at the inlet. The parameters of each three-element Windkessel model were manually tuned to give typical flow and pressure waveforms at the outlets (Xiao et al. 2014). The values of these parameters are given in Table 4.

Figure 5 shows pressure and flow wave forms at selected outlets as well as visualisations of pressure and flow in the aorta at peak systole. The results show physiologically realistic ranges for flow, pressure and flow splits between different vascular regions.

#### Dissected aorta

Following the simulation of the undissected model, flow through the dissected model was investigated using the same inlet and outlet boundary conditions as in the preceding analysis. From the three 2D PC-MRI acquisitions in the descending aorta, it was known that mean total flow (for both true and false lumina) ranges between of 55.6 and $50.3\,\hbox {cm}^{3}$/s. It was found that the simulated flow down the descending dissected aorta lied within these limits without having to change any of the inflow and outflow boundary conditions relative to the undissected model.

**Table 4:** RCR parameters tuned to produce physiologic flow and pressure waveforms for the undissected aortic model

	$\hbox {R}_{\mathrm{p}} [\hbox {g}/(\hbox {mm}^{4}\,\hbox {s})]$	$\hbox {C} (\hbox {mm}^{4}\ \hbox {s}^{2}/\hbox {g})$	$\hbox {R}_{\mathrm{d}} [\hbox {g}/(\hbox {mm}^{4}\hbox { s})]$
RCCA	0.80	6.10	4.53
RSCA	0.13	7.64	1.89
LCCA	0.78	6.14	4.40
LSCA	0.12	7.87	1.72
Hepatic	0.24	1.15	4.06
Splenic	0.14	2.04	2.30
M Coeliac	0.23	1.18	3.95
SMA	0.13	3.87	1.15
R Renal 1	0.48	2.37	1.94
R Renal 2	1.11	1.19	4.45
L Renal	0.32	3.34	1.30
IMA	0.68	0.73	6.13
M Sacral 1	0.54	0.52	9.04
M Sacral 2	0.78	0.36	13.12
L Int Iliac	0.10	0.15	3.87
R Int Iliac	0.13	0.15	5.15
L Ext Iliac	0.06	0.49	2.49
R Ext Iliac	0.06	0.55	2.18

**Fig. 6:**
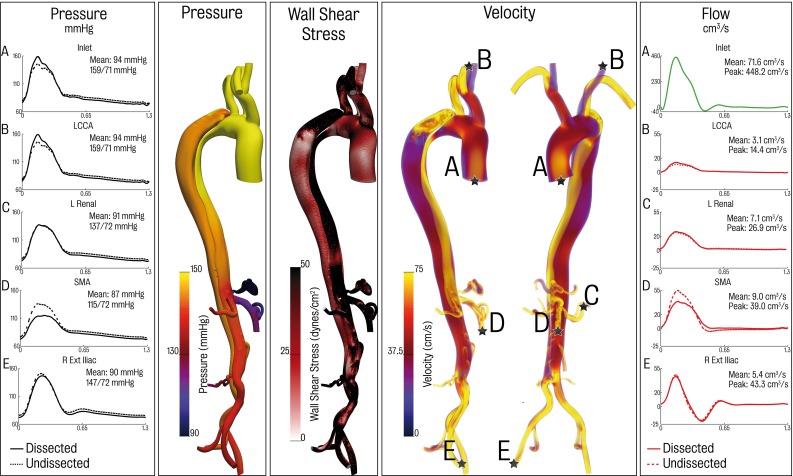
Visualisations of pressure, wall shear stress and velocity at peak systole for the baseline dissected model at peak systole. Shown also are comparative pressure and flow waveform plots at the inlet, left common carotid, left renal, superior mesenteric artery and right external iliac for both the undissected and dissected models

**Fig. 7:**
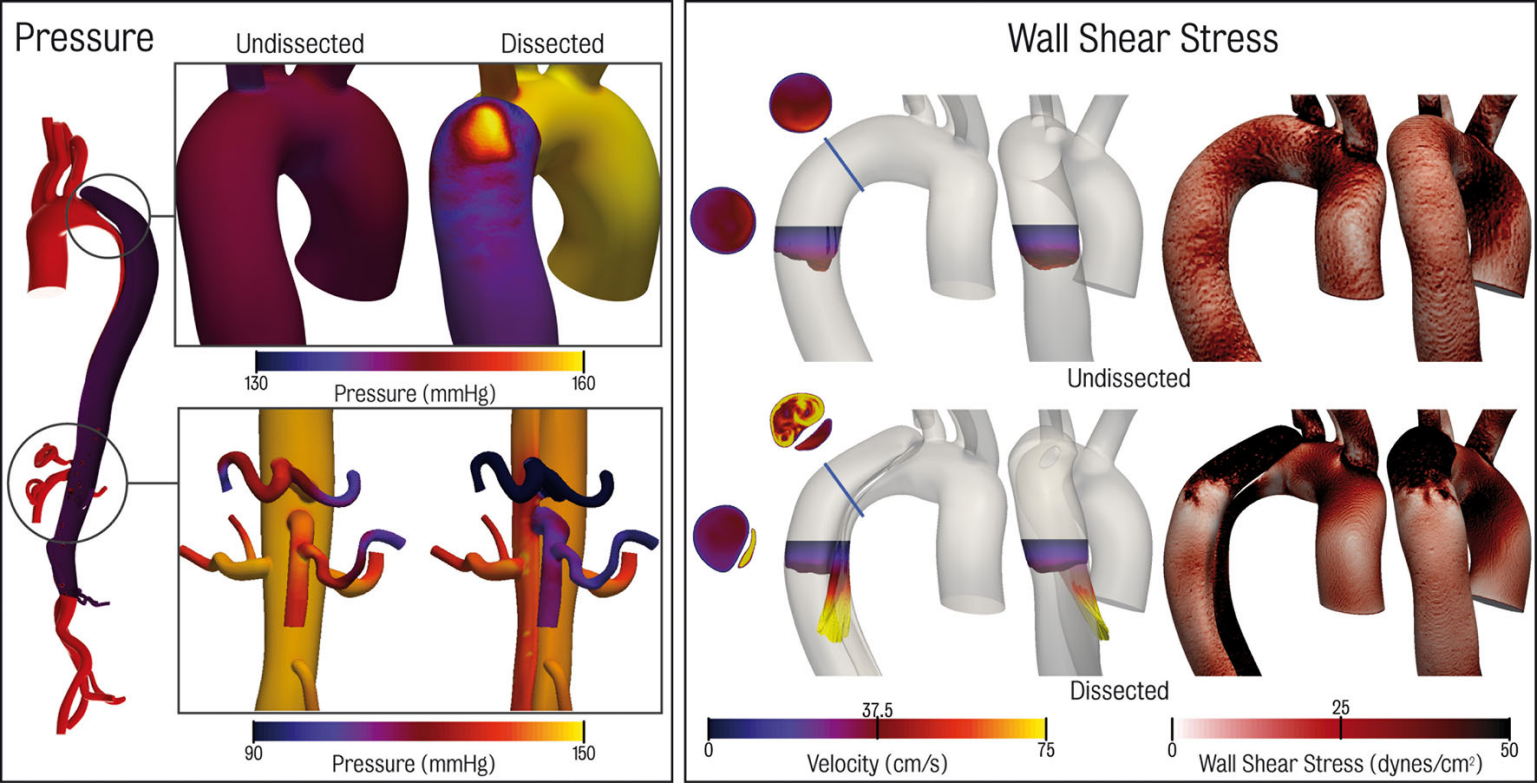
Localised regions of haemodynamic change in the presence of dissection comparing pressure and wall shear stress at peak systole between the dissected and undissected models

Figure 6 shows haemodynamics in the dissected aorta at peak systole. Significant changes are apparent relative to the results depicted in Fig. 5. In terms of pressure at the different branches, there is a clear increase in pulse pressure at the aortic inflow and the upper branches. The systolic and diastolic left carotid pressure are 146/75 mmHg for the undissected case and 159/71 mmHg for the dissected case. The mean pressure for the ascending vessels remains relatively constant. Flow is increased to these branches. Specifically, flow to the left common carotid increased by 14.8 % in the dissected case. The vessels in the distal descending aorta experienced drops in both mean and pulse pressure. For instance, the right external iliac had a mean and pressure of 94 mmHg and systolic and diastolic pressures of 151/76 mmHg in the undissected case, whereas the mean, systolic and diastolic pressures for the dissected case are 90 and 147/72 mmHg. Several other outlets experienced changes in pressure and flow, for example, in the SMA mean pressure dropped by 6.5 % and mean flow by 5.3 %. Flow to the vessels branching from the coeliac trunk dropped by a total of 13.5 % and pressure by 13.6 %.

Locally, there are significant alterations in haemodynamics, particularly around the entry tear, true lumen and coeliac trunk regions. Some of these changes are highlighted in Fig. 7. The spatial distribution of pressure in the dissected model at peak systole around the entry tear ranges between 58.6 and 161.0 mmHg. This range was much smaller in the corresponding region of the undissected model: 92.2–148.0 mmHg. The peak systolic pressure gradient along the length of the true lumen is larger than that seen in the corresponding location of the undissected aorta: from 160 mmHg at the entry tear to 135 mmHg at the exit tear in the dissected model. Correspondingly, the undissected model has a pressure gradient of 5 mmHg in the descending aorta with pressures in the range of 145–140 mmHg. Pressure in the proximal portion of the dissection is generally higher in the true lumen than in the false. The reverse trend is observed in the distal portion. This pattern has also been noted in other studies (Tse et al. 2011; Alimohammadi et al. 2014). An area of marked increased pressure (over 160 mmHg) is observed where flow through the entry tear impinges on the wall of the false lumen. This localised region of high blood pressure on the structurally compromised false lumen could lead to enlargement and potential rupture (Tsai et al. 2007; Chen et al. 2013).

The dissection case shows large true lumen peak velocities in the range of 70 cm/s in the coeliac trunk region where there is a large flow demand. Distal to the coeliac trunk region, there is a significant drop in peak velocities. The computed values of 35 cm/s are comparable with those seen in the undissected case.

Highly complex flow patterns are observed in the false lumen in the entry tear region. A dramatic increase in time-averaged wall shear stress (TAWSS) from approximately 5 dynes/$\hbox {cm}^{2}$ in the undissected case to over 100 dynes/$\hbox {cm}^{2}$ in the dissected case is observed. A more moderate increase in true lumen TAWSS is observed in the coeliac trunk region, going from 5 to 10 dynes/$\hbox {cm}^{2}$ in the undissected case to over 20 dynes/$\hbox {cm}^{2}$ in the dissected case.

**Fig. 8:**
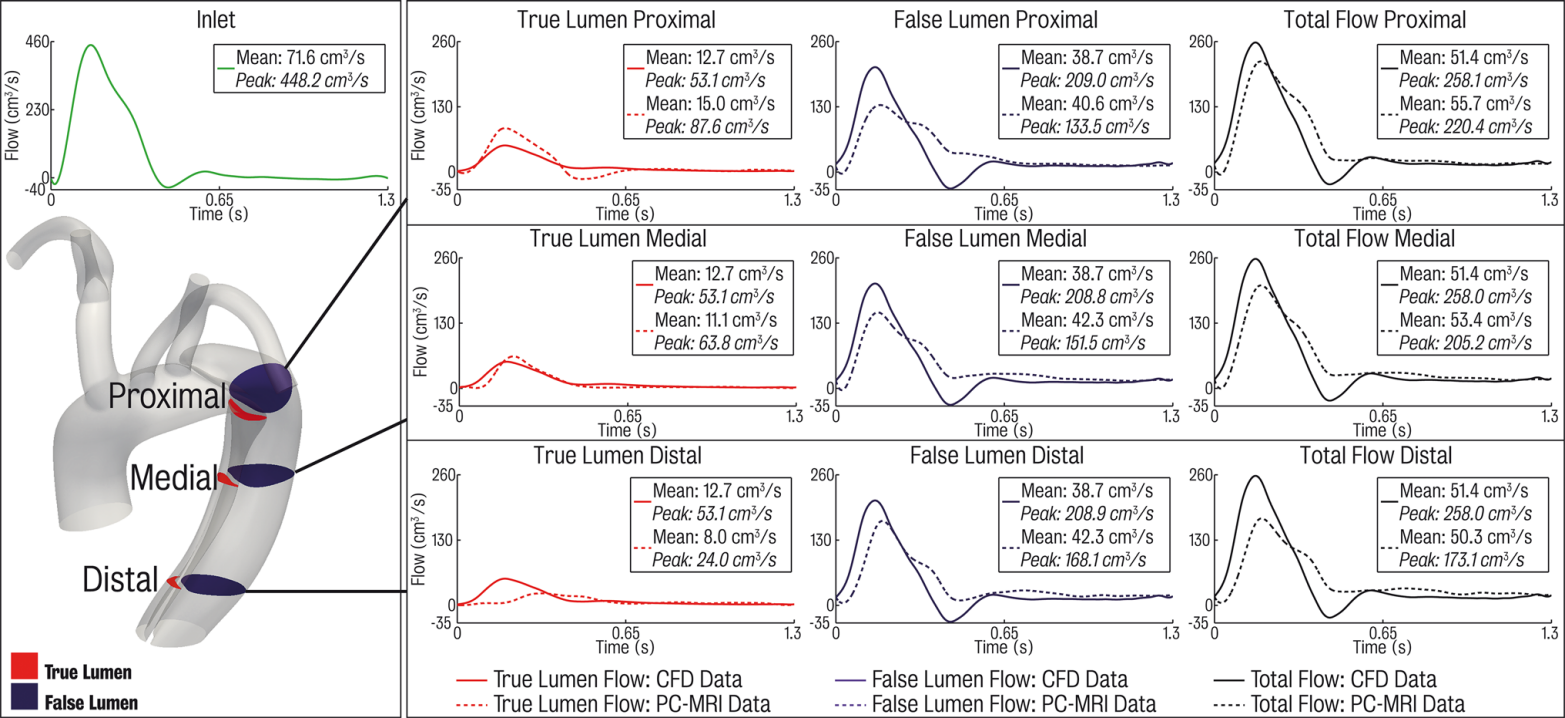
Comparison between 2D PC-MRI and CFD simulation data for the true lumen, false lumen and total flow. True lumen is shown in *red*, false lumen in *purple* and the total flow in *black*. CFD data are shown by the *solid lines*, while 2D PC-MRI data are presented in the *dashed lines*

#### Comparison between computed and measured flows in the descending aorta

In this section, we compare the computed flows in the true and false lumina with those measured using PC-MRI at three different locations in the descending thoracic aorta. For clarity, these shall be referred to as proximal, medial and distal locations. Figure 8 shows quantitative comparisons of PC-MRI and simulation data at each location.

There is good agreement in mean flows in true and false lumina between simulation and imaging data. The MRI gives a total flow (e.g. sum of false lumen and true lumen flow) at each location of 55.6, 53.8 and 50.3 $\hbox {cm}^{3}$/s respectively. The computation predicts a uniform value for the total flow of 51.4 $\hbox {cm}^{3}$/s at each location, resulting in relative errors between measurement and simulation of $-7.6$, $-4.5$ and $+2.2\,\%$, respectively. It is important to note that this good agreement in total mean flow between data and simulations was not imposed in any way, but rather the result of properly tuned outflow boundary conditions in the remaining branches of the model as these dictated the flow splits to the different branches, as well as a realistic reconstruction of the aortic anatomy. On average, the false lumen carried 79 % of the total descending aortic flow according to the MRI, whereas in the simulation this value was 76 %.

Examining each lumen individually, MRI false lumen mean flows at each location are 40.6, 42.5 and 42.4 $\hbox {cm}^{3}$/s, whereas the simulations produce a nearly uniform mean value of 38.7 $\hbox {cm}^{3}$/s at all three locations, resulting in relative errors of $-4.7$, $-8.9$ and $-8.7\,\%$, respectively. Considering the true lumen, MRI mean flows are 15.1, 11.1 and 7.9 $\hbox {cm}^{3}$/s at the location, while the computations show a constant value of 12.7 $\hbox {cm}^{3}$/s for all three locations and relative errors of $-15.9$, $+14.4$ and $+60.8\,\%$, respectively. The larger relative errors in true lumen flow can be explained by the smaller mean values recorded at this location. On average, MRI data show the true lumen carries 21 % of total flow, whereas in the simulation this value is 25 %. It is important to note that PC-MRI data were acquired with single VENC, but as peak velocities were significantly lower in the false lumen than in the true lumen (in the order of 50 % less), true lumen and false lumen flows may have differed somewhat from what was recorded.

Overall, the small differences in flow between the three locations are to be expected due to the close proximity of the different slices (11 cm between proximal and distal slices) and the apparent lack of secondary connecting tears or branching vessels between the proximal and distal slices. The increase in measured false lumen flow from the proximal to the distal location (40.6 vs. $42.3\,\hbox {cm}^{3}/\hbox {s}$) could suggest the presence of a connecting tear. However, this could not be located in neither the CT data nor the 4D-PC-MRI data. In the simulation data, this lack of connecting tears results in identical flows at each location due to the rigid wall and blood incompressibility assumptions. In Sect. 3.3, we study the impact that additional connecting tears between lumina may have in the aortic haemodynamics.

**Table 5:** Tuned parameters of the heart model for the dissected and undissected models

	Dissected model	Undissected model
Period (s)	1.3	1.3
End diastolic volume $(\hbox {mm}^{3})$	130,000	130,000
Unstressed volume $(\hbox {mm}^{3}$)	0.0	0.0
Preload (g/$\hbox {mm}^{2}$)	533.320	533.320
Time to maximum elastance (s)	0.410	0.420
Time to relaxation (s)	0.205	0.210
Maximum elastance (g/($\hbox {mm}^{4}\hbox { s}^{2}$))	0.460	0.400
Minimum elastance (g/($\hbox {mm}^{4}\hbox { s}^{2}$))	$4.102\times 10^{-3}$	$4.102 \times 10^{-3}$
Aortic valve resistance [g/($\hbox {mm}^{4}\hbox { s}$)]	$1.0 \times 10^{-5}$	$1.0 \times 10^{-5}$
Aortic valve inductance [g/$\hbox {mm}^{4}$]	$1.0 \times 10^{-5}$	$1.0 \times 10^{-5}$
Ventricular resistance (s/$\hbox {mm}^{3}$)	$5.0 \times 10^{-7}$	$5.0 \times 10^{-7}$
Mitral valve Resistance [g/($\hbox {mm}^{4}\hbox { s}$)]	$3.9453 \times 10^{-7}$	$3.9453 \times 10^{-7}$
Mitral valve Inductance (g/$\hbox {mm}^{4}$)	$1.334 \times 10^{-5}$	$1.334 \times 10^{-5}$

Despite the mean flows being relatively well matched between PC-MRI and simulated data, there exists a greater discrepancy when considering the systolic and diastolic range at each location. In the true lumen at two of the three locations, the peak-to-peak range of flows from the simulated data underestimated that of the PC-MRI data, while in all three false lumen locations the amplitude of the simulated waveforms is larger than that of the PC-MRI data. PC-MRI data for the true lumen have a systolic/diastolic range of $-13.8$/87.6, 0.8/63.4 and 0.6/24.0 $\hbox {cm}^{3}$/s at the respective locations, while the simulated data at the same locations have a range of 1.9/53.1, 1.9/53.1 and 1.9/53.1 $\hbox {cm}^{3}$/s. In the false lumen, the PC-MRI data have a range of 133.5/10.3, 151.5/14.9 and 168.1/10.1 at the respective locations, while the corresponding simulated data show ranges of 209.0/$-32.7$, 208.8/$-32.8$ and 208.9/$-32.8$. These discrepancies may be explained by the lack of compliance in the rigid 3D aortic model. In reality, the vessel walls are compliant and the septum deforms significantly, especially in an acute setting. The arterial compliance enables the vessel to accommodate part of the blood volume during systole, reducing the peak flow rates through a given section. In early diastole, the stored blood volume is released, increasing minimum flow during diastole. A rigid 3D model cannot capture this effect however, and the only compliance in the computational model is provided by the capacitor component in the 0D Windkessel model coupled to each outlet. This is supported by the observed of higher peak-to-peak flows of the simulated to PC-MRI data and is expected when using a rigid model.

### Study 2: Analysis of the impact of the flap on left ventricular function

In this study, a lumped-parameter heart model was coupled to the inlet face of the dissected and undissected models to examine changes in stroke work induced by the dissection. The heart model enables the quantification of left ventricular volume and pressure throughout the cardiac cycle. From these data, a pressure volume loop can be generated to quantify the cardiac workload. The cardiac output, measured with MRI in the dissected aorta, was assumed to be identical for both dissected and undissected cases. This is a reasonable first approximation, as the heart is known to adjust to changes in afterload to maintain a relatively constant output that satisfies the metabolic demands (Lau and Figueroa 2015). The outflow boundary conditions were kept unchanged. The elastance function characterising the performance of the heart model was adjusted in both cases to match the measured flow waveform at the level of the aortic root. The iterative process sought to match mean cardiac output, systolic cardiac output, cardiac cycle and length of systolic phase. The adjusted parameters were maximum elastance and time to maximum elastance (Table 5). Iterations on maximum elastance and time to maximum elastance parameters stopped when the obtained maximum and mean cardiac output were within 5 % of the measured PC-MRI values.

**Fig. 9:**
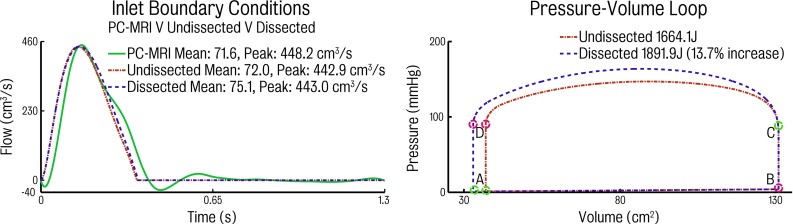
*Left* inflow waveforms obtained with PC-MRI and heart model. *Right* pressure–volume (*PV*) loops generated via using the heart model for both the undissected and dissected simulations. The PV loops illustrate the mitral valve opening (*A*), the diastolic ventricular filling (*A*–*B*), mitral valve closing (*B*), isovolumetric contraction (*B*–*C*), aortic valve opening (*C*), ejection (*C*–*D*), aortic valve closing (*D*), followed by isovolumetric relaxation (*D*–*A*). The *area enclosed by the PV loop* represents the left ventricular stroke work. There is an increase in the stroke work of 13.7 % in the presence of dissection

The left panel of Fig. 9 shows a good match in the waveforms generated via the 0D heart model and the physiological PC-MRI data. The heart model waveforms for both dissected and undissected cases lack the slight early diastolic back flow observed in the PC-MRI data. This is a direct consequence of the way the aortic valve is modelled in the 0D heart model (see Fig. 4): the diode representing the function of the aortic valve closes instantly as soon an adverse pressure gradient between the left ventricle and the aorta is detected. This effectively precludes back flow when using the heart model.

The right panel of Fig. 9 shows the PV loops for both dissected and dissected cases. The four distinct phases of the cardiac cycle (ventricular filling, isovolumetric contraction, ejection and isovolumetric relaxation) as well as the aortic valve opening and closing can be observed in the diagrams. The estimated left ventricular work load, quantified by the area enclosed by the plot, shows a significant increase (13.7 %) in the dissected case work (1.9 kJ), relative to the work performed by the heart in the undissected anatomy (1.7 kJ). The increased in work is the result of an increase in maximum elastance during peak systole due to the higher afterload in the dissected case. The presence of the septum therefore makes the heart work harder to produce the same cardiac output as in the undissected case.

### Study 3: Sensitivity study of the impact of secondary entry tears

**Fig. 10:**
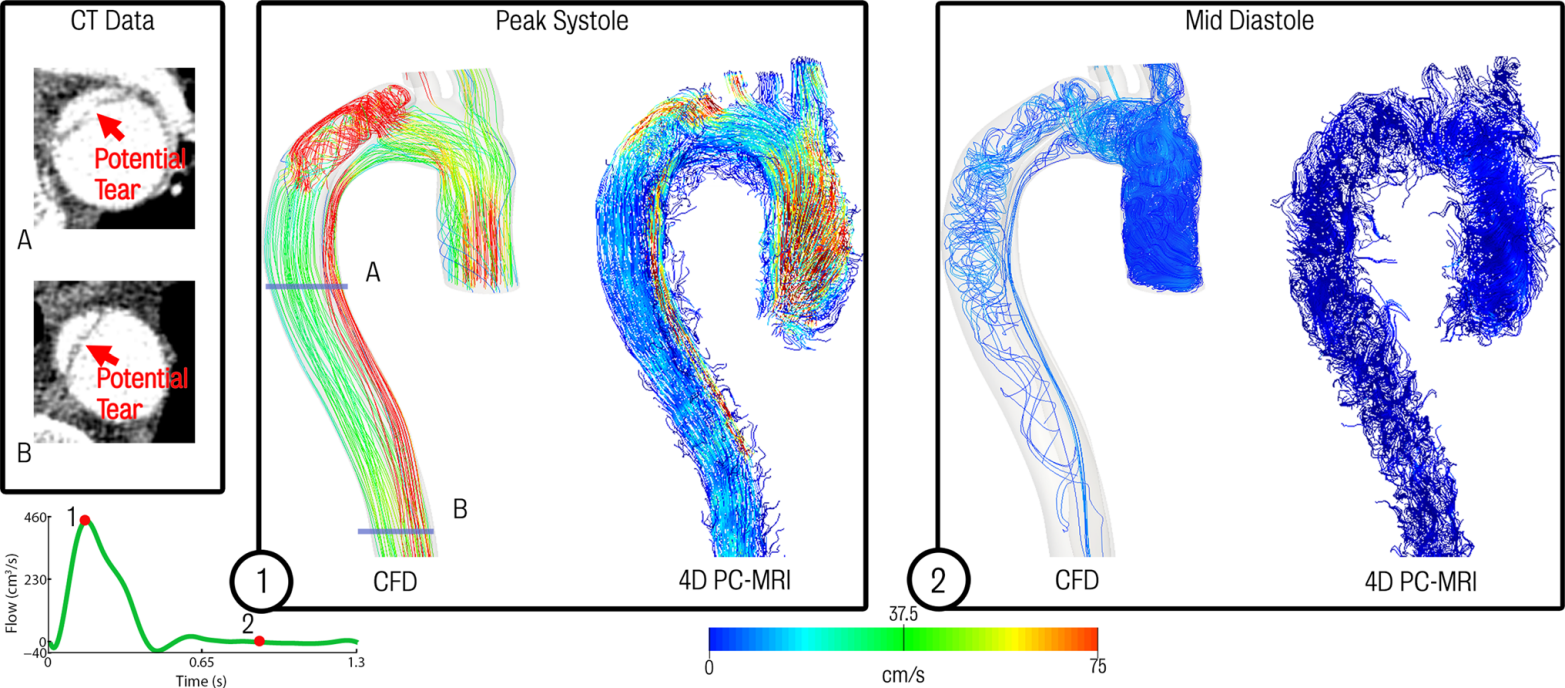
Comparison of CFD data and acquired 4D PC-MRI flow data at peak systole and mid-diastole. Also shown are the CT image data at two locations showing suspected secondary tears which were not apparent on the 4D PC-MRI data

While the location of some of the tears appeared to be clear in the CTA (see Fig. 10, left), the tear location could not be confirmed by the 4D PC-MRI data, as there was no distinct change in the velocity field that would indicate a connection between the true and false lumina (see Fig. 10, centre and right panels). These panels also show a comparison between CFD and 4D PC-MRI streamlines at peak systole and mid-diastole. Good qualitative agreement between patterns in the velocity field in data and simulation can be observed: for example, high velocities around the main entry tear and in the true lumen are apparent in both imaging and simulation data. An important question therefore remains, do secondary tears have a significant impact on dissection haemodynamics and is there a better agreement between clinical data (e.g. MR-derived flow) and CFD results when secondary tears are included?

**Fig. 11:**
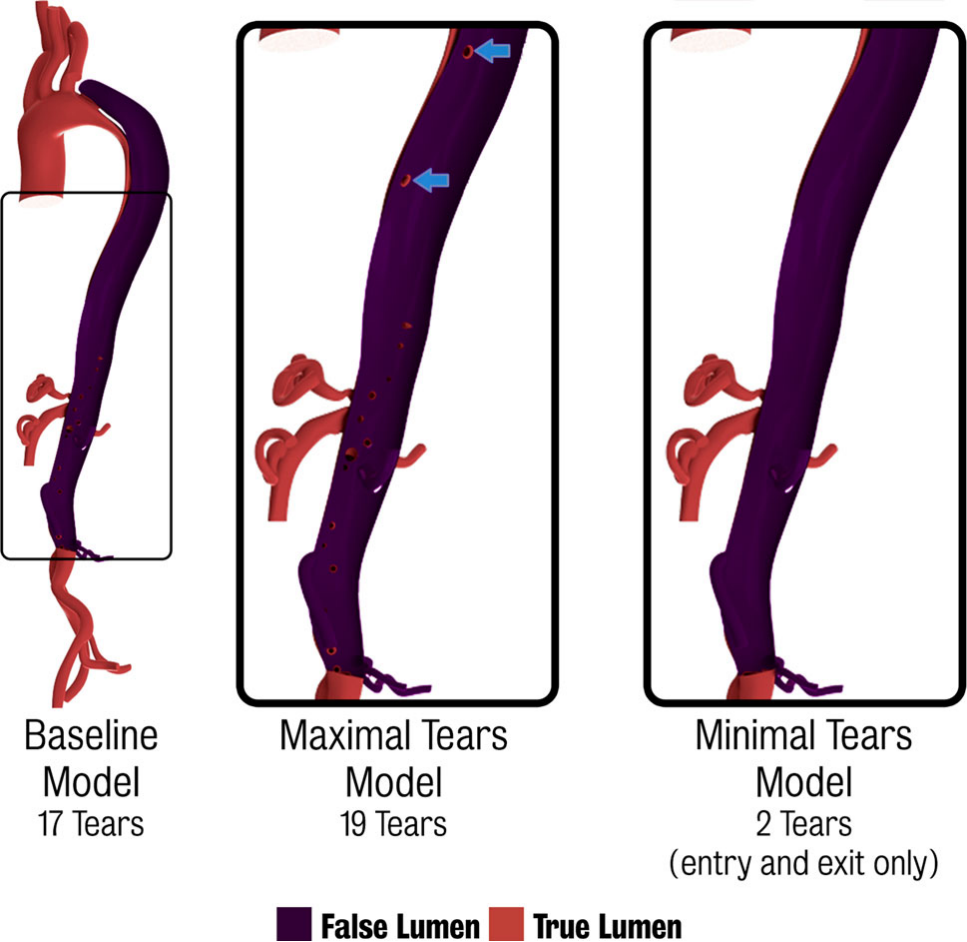
*Left* baseline dissected model featuring a total of 17 tears. *Centre* “maximal tears” model with two additional tears in the descending aorta evident in the CT data but not in 4D PC-MRI. *Right* “minimal tears” model where all but the primary entry and exit tears were removed

A study was thus designed to investigate the influence of secondary tears on aortic dissection haemodynamics. Two modifications of the 17-tear baseline dissected model were constructed. The first is a “maximal tears” model in which two additional tears in the descending aorta were included. These tears, while evident on the CT data, were not seen in the 4D PC-MRI data. The second is a “minimal tears” model containing only the primary entry and exit tears (see Fig. 11). The final adapted finite element mesh sizes for all three models are given in Table 6.

**Fig. 12:**
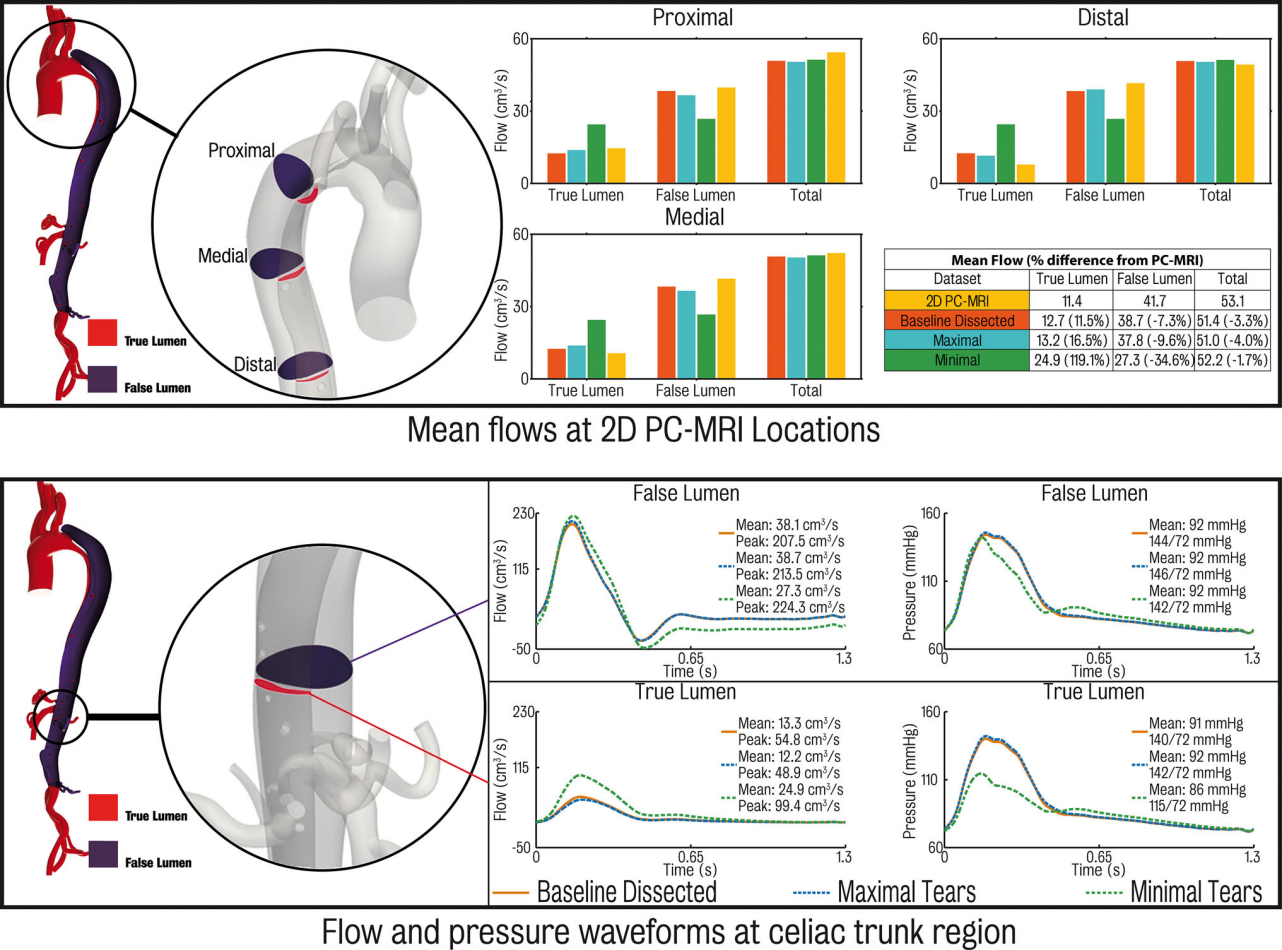
*Top* comparison of computed mean flows and 2D PC-MRI data for the proximal, medial locations of the descending thoracic aorta. The table gives average values of error over the three locations. *Bottom* computed pressure and flow waveforms for each of the three dissection models at a location just proximal to the coeliac trunk

Comparisons between the 2D PC-MRI data acquired and CFD results at each of the three locations (proximal, medial and distal) for the three dissection models are shown in the top panel of Fig. 12. The computed total flow (defined as the sum of true and false lumen flow) shows a good comparison with the 2D PC-MRI data for all three models, with mean simulation errors ranging from $-3\,\%$ (maximal tears) to $-1.4\,\%$ (minimal tears). However, when comparing flow through each individual lumen, the minimal tears case shows larger errors than the baseline and maximal tears models: 122.6 % mean error in the true lumen and $-34.7\,\%$ error in the false lumen. The baseline and maximal tears models show similar results, and match the 2D PC-MRI data more closely.

The bottom panel of Fig. 12 shows a comparative of flow and pressure waveforms in the region proximal to the coeliac trunk for each of the models. Note that no patient-specific data were acquired in this region, and this comparison is presented to highlight the differences between each simulation. The results show that the baseline dissected and maximal tears cases are relatively comparable, whereas the pressure and flow waveforms in the minimal tears case are significantly different. These differences are more pronounced in true lumen, where large changes in peak ($+81.4\,\%$) and mean ($+87.3\,\%$) flow and peak pressure ($-17.8\,\%$) are observed relative the baseline dissected case. Tears in the septum allow pressure between the true and false lumina to equalise. The differences observed in the true lumen of the minimal tears case are likely a combined effect of the flow demand of the branching vessels in this region having to be supplied solely by the lumen they branch from resulting in increased flow in the true lumen, and Poiseuille’s law, as the effective cross-sectional area communicating blood to the true lumen decreases due to the absence of the communicating tears and results in a pressure drop.

**Table 6:** Final mesh sizes for each dissected model

Model	Mesh size($\times 10^{6}$)
Baseline dissected	1.8 nodes/11.8 elements
Maximal tears	2.4 nodes/13.2 elements
Minimal tears	1.8 nodes/11.5 elements

These results suggest that in this case some tears do indeed exit in the trunk region given that the minimal tears case shows results clearly different from the baseline and maximal tears, and with higher errors compared to the 2D PC-MRI data. The issue however still remains, as these small secondary tears in a thin and highly distensible dissection flap prove to be very difficult to image.

## Discussion

In this study, we used multiple sources of medical image data (CT; 2D PC-MRI velocity data in the true and false lumina and ascending aorta; and 4D PC-MRI) to inform our computational analysis of aortic dissection. The combination of highly resolved CT and 2D PC-MRI data has allowed, for the first time, the creation of a highly detailed, multi-branch geometric model of the dissected morphology featuring 17 branches. The 2D PC-MRI flow data were used to tune both the inflow and outflow reduced-order models coupled to the 3D domain, and to validate the simulation results.

**Fig. 13:**
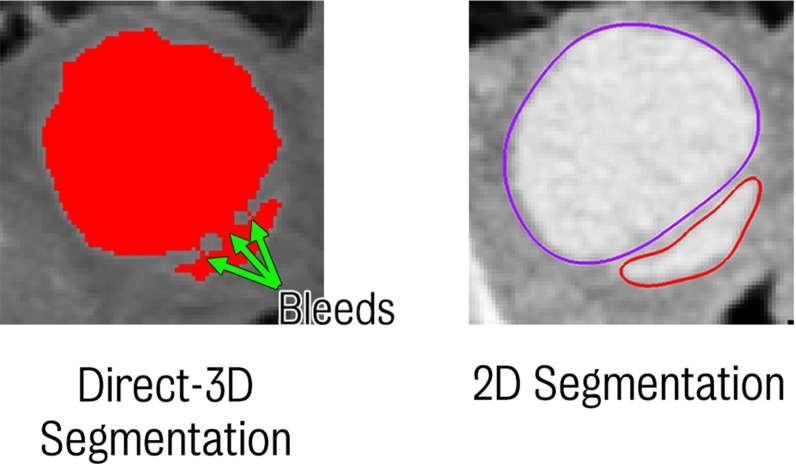
Direct 3D segmentation (*left*) illustrating typical “bleeds” between the true and false lumina through the relatively thin and poorly defined septum. For comparison, a typical 2D segmentation in the same plane is shown (*right*) where contours may be automatically created and corrected manually if necessary

Building a geometric model of the aortic vasculature involves segmenting the patient’s image data to obtain the volume of interest where the blood flow simulation is to be performed. There are several powerful 3D automatic and semiautomatic segmentation tools such as Seg3D (CIBC 2015), MITK (Wolf et al. 2005) and ITK-Snap (Yushkevich et al. 2006) that can perform this task with relatively little effort from the user. However, automatic segmentation may be difficult to apply on images with low resolution or poor signal-to-noise ratio, circumstances unfortunately present in aortic dissection imaging. Indeed, the small size and motion of the septum often make it difficult to delineate the boundaries between true and false lumina. It was found that, after trying several different tools, automatic 3D segmentation methods produced numerous artefacts in the form of “bleeds” between the two lumina (see Fig. 13). A significant amount of time was spent trying to manually correct these artefacts, a task that is non-practical as it was significantly more time-consuming than the segmentation process itself. It was therefore concluded that in cases of aortic dissection, a 2D segmentation approach is preferable. This segmentation approach, although requiring a larger degree of user intervention (i.e. it is less automatic), is more robust in situations of poorly defined features in the image data as the lofting method interpolates the surface between each contour which may be defined at locations where said features are clearly visible and also allows for human judgement to be employed in regions where features may be obscure.

Previous CFD studies in aortic dissection have been somewhat limited due to the assumptions adopted to determine inflow and outflow boundary conditions. For instance, most studies have used flow data acquired from healthy subjects (Tse et al. 2011; Chen et al. 2013; Alimohammadi et al. 2014). Dissection patients are often prescribed the antihypertensive therapies such as beta blockers and morphine sulphate and this may have associated issues. The therapies seek to maintain normal blood pressure, thereby reducing aortic wall stress in dissection patients (Elefteriades et al. 1999; Erbel et al. 2001; Nienaber and Eagle 2003; McGee et al. 2005; Coady et al. 2010). However, cardiac output is often also reduced via reduction in left ventricular contractility. Therefore, assuming healthy inflow conditions may overestimate actual inflow conditions (Chen et al. 2013) in dissection patients. Our paper has used patient-specific data at a number of locations to inform and validate the model. Similarly, with regard to outflow boundary conditions, most studies have either imposed pressure and/or velocities acquired from healthy subjects or have applied zero pressure conditions (Tse et al. 2011; Karmonik et al. 2011a; Chen et al. 2013). In this study, we have used tuned lumped-parameter Windkessel models to represent the resistance and compliance of the distal vasculature.

We performed three studies with two main overriding objectives: i) to validate our computational results against subject-specific flow data, and ii) to obtain a deeper understanding on the alterations in aortic haemodynamics induced by the dissection.

In the first study, a comparison between haemodynamics in a subject-specific aortic dissection model and a hypothetical baseline normal (undissected) aorta was performed. The undissected model was created by virtually removing the intimal flap of the dissected aorta, assuming that no remodelling (e.g. false lumen expansion) had occurred due to the acute nature of the dissection. Assuming identical cardiac output for both the dissected and undissected models, our results show significant differences between the models and give an estimation of the haemodynamic changes that occurred for this patient with the onset of the dissection. There is an increase in the ascending aortic pulse pressure in the dissected model (159/71 mmHg, pulse 88 mmHg) compared to the undissected model (146/74 mmHg, pulse 72 mmHg). These differences are consistent with the larger afterload induced by the dissection septum compared to the baseline model. In the dissected case, the true lumen exhibits a much larger pressure gradient in the descending aorta when compared to the undissected model, particularly in the region proximal to the coeliac trunk. This larger pressure gradient is associated with higher peak velocities and larger wall shear stress. The section distal to the coeliac trunk region shows a drop in both the true lumen pressure gradient, mean pressure, and reduced peak velocity and wall shear stress. Local regions of high velocities and wall shear stress are observed where flow through connecting tears impinge on the true lumen wall. Flow through the main entry tear creates a jet that impinges on the false lumen wall, creating a region of highly complex flow, increased pressure and WSS. As Tolenaar et al. (2013a) hypnotised, this region of high pressure in the false lumen may indicate an area of concern as a region where aortic growth is probable. The false lumen carries a greater proportion of descending aortic flow and is significantly larger than the true lumen. The false lumen exhibits a more homogenous pressure gradient along its length, with lower velocities and lower wall shear stress than the true lumen.

In a second study, we investigated the changes in left ventricular workload in aortic dissection. This was done by using a 0D lumped-parameter heart model coupled to the image-based aortic model. Using this approach, left ventricular function can be obtained from the interactions between the heart model and the afterload provided by the aorta and the Windkessels. Left ventricular volume and pressure can be recorded, and a PV loop obtained. The intimal flap and more complex flow patterns in the aorta result in higher energy losses and an effective increase in the hydraulic resistance felt by the heart. The heart model was tuned using values derived from Lau and Figueroa (2015) to match the acquired 2D PC-MRI flow data and then adjusted to produce the same cardiac output in the undissected case. Our results showed that there is an increase in left ventricular stroke work of 13.7 % in the dissected case compared to the undissected case for the same cardiac output. This increase is equivalent to adding a full day of work per week. This is therefore an important consideration to observe in the management of chronic dissection patients, where conservative medical management is often adopted as a long-term strategy.

Previous aortic dissection CFD studies have typically used models consisting of a single entry and/or exit tear (Karmonik et al. 2008; Rudenick et al. 2010; Karmonik et al. 2011b, a; Heim et al. 2013). The effect of varying size and location of entry and exit tears in dissection has been studied (Tsai et al. 2008; Karmonik et al. 2011a; Rudenick et al. 2013), and observational studies have examined the correlation of the number of communication tears and rate of growth of dissection (Tolenaar et al. 2013b). Generally, the relatively small secondary tears have not been included in CFD studies of dissection. For the third study, we have tried, for the first time, to quantify the effect of secondary tears and found that these tears play a significant role in dissection haemodynamics. In our study, while the size of the individual tears was not altered, the number of connecting tears in both the proximal and distal parts of the dissection was. In other words, the total cross-sectional area available for communication between lumina changes as a result of the increase in the number of communicating tears. Our results showed that multiple tears along the length of the dissection result in an equalisation of pressure between the lumina. An increase in false lumen flow was observed when more communicating tears are present. This would seem to be consistent with other clinical and experimental studies which have suggested that these secondary tears have a significant impact on the haemodynamics. Tolenaar et al. (2013b) showed that patients with a single entry tear had significantly poorer outcomes than those with multiple tears while Rudenick et al. (2013) showed that small connecting tears resulted in a larger pressure difference between the lumina with higher pressures observed in the true lumen, while larger tears resulted in an equalisation of pressure between the lumina. The study also showed that the true lumen receives a larger proportion of flow in the case of smaller tears. Berguer et al. (2014) showed that the pressure differential between the true and false lumina is inversely proportional to the cross-sectional area of the exit tear. From the maximal tears results, it would also appear the inclusion of the two tears in the thoracic aorta had little effect on the pressures or flows in the lumina relative to the baseline case, and it may therefore not be necessary to include all connecting tears to ensure relatively accurate modelling of the dissection haemodynamics.

We have also shown that the number of connecting tears results in significant changes in both pressure and flow particularly in the true lumen. This may be due to the high flow demand of the branch vessels around the coeliac trunk: secondary tears may facilitate the required flow to satisfy this demand. In the absence of secondary tears, this demand must be satisfied by the true lumen entirely. It is however clear that these tears are small in size and therefore difficult to identify with current imaging technology.

### Limitations and future work

#### Imaging limitations

The primary imaging modality used to create the aortic dissection computer model was non-gated contrast-enhanced CT. It has been observed that non-gated CT may contain pulsation artefacts that can result in the generation of an artificial intimal flap (Qanadli et al. 1999; Flohr et al. 2002). There are inherent disadvantages to gated CT such as higher radiation dose and longer scan time. However, gating does allow for the capture of the septum at the same phase in the cardiac cycle, thereby eliminating motion due to pulsation. Imaging the septum in such a way should eliminate the majority of motion artefacts and allow the reconstruction of a more accurate representation of the geometry.

**Fig. 14:**
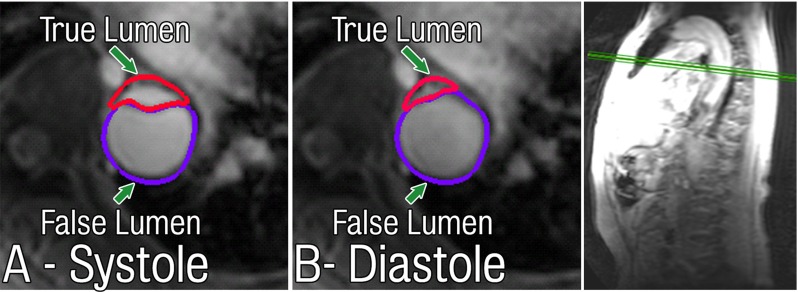
2D MRI showing distention of the dissection flap between peek systole (**A**) and mid-diastole (**B**)

It is generally desirable to have higher spatial resolution for both CT and MRI data when performing image segmentation as low spatial resolution limits the size of features that may be included in the computer model, and, in this instance, made identification of the relatively small secondary tears difficult. Twenty-five images were acquired for both 2D and 4D PC-MRI data sets resulting in a temporal resolution of 52 ms. When acquiring PC-MRI data, higher temporal resolution may enable better observation of the septum motion. Higher temporal and spatial resolution of 4D PC-MRI should allow for flow patterns through tears to be more readily observed.

The 2D PC-MRI data were acquired at three locations in close proximity in the thoracic region of the aorta. A greater number of 2D PC-MRI scans capturing flow in the coeliac and iliac regions may have enabled a better, more accurate tuning of the outflow Windkessel models. Furthermore, dual VENC (velocity encoding) in the descending aorta would have increased the fidelity of the reconstructed true lumen and false lumen flows since significantly different maximum velocities are found in the two lumina.

The results indicate that inclusion of the difficult to image connecting tears is necessary for accurate modelling. Arterial spin labelling (ASL) is an MRI technique which allows the tracking of labelled blood over time (Wong 2014). This technique could be used in dissection patients to identify secondary connecting tears by detecting the transfer of labelled blood between the lumina. Lastly, initial results using black-blood imaging techniques have yielded excellent results in imaging the intimal flap accurately. This technique avoids the exposure to radiation of CT we hope to use this technique for image acquisition in cases of aortic dissection.

#### Pressure data

In this study, we aimed to reproduce physiologic values of pressure in the thoracic aorta since we did not have subject-specific pressure data. This is an important limitation as the pressure in true and false lumina may differ significantly depending of the size and number of connecting tears (Rudenick et al. 2013). In our paper, the large size of the connecting tears (13 mm proximally and 9.3 mm distally) makes the differences between true and false lumina pressure modest, and therefore, the lack of pressure data may be less critical. In future studies, patient-specific pressure data using soft, pliable wires with a high-fidelity sensors will be collected at multiple locations in the aorta from patients during planned endovascular procedures. These pressure data will provide a gold standard for the simulated CFD pressures.

#### Modelling limitations

The main limitation in the modelling approach of this paper is the rigid wall assumption. Imaging of the intimal flap using 2D MRI techniques shows portions of the flap that undergo significant deformations during systole (see Fig. 14). Other studies have shown that the true lumen experiences expansion and forward flow during systole, and an almost complete collapse during diastole due to pressure fluctuations. This may increase the risk of end-organ ischaemia. False lumen flow can be seen to be delayed, absent or even reversed, depending on the geometry configuration of the tear and the degree of communication between the true and false lumina (Erbel et al. 2001).

The mismatch in amplitude between the computed and measured flow wave forms observed is due to the lack of compliance in both the aortic wall and intimal flap of the computer model. This compliance can only be represented via a suitable fluid–structure interaction (FSI) method. A FSI method will however increase the computational cost of the simulations significantly. In recent years, non-boundary- fitted methods pioneered by Peskin (1972) have been developed to represent the dynamics of thin structures embedded in a fluid. These methods, such as the Fictitious domain method (Glowinski et al. 1994), the mortar element method (Baaijens 2001) and the immersed structural potential method (ISPM) (Gil et al. 2010) allow for the imposition of the necessary kinematic (no-slip) and dynamic (balance of forces) compatibility conditions without resorting to computationally more expensive methods that require continuous update of the computational grid as in boundary-fitted Arbitrary Lagrangian Eulerian (ALE) formulations (Nordsletten et al. 2010a, b).

When comparing haemodynamics in the dissected and undissected models, two main assumptions were made. First, given the acute nature of the dissection, we assumed that no false lumen remodelling had taken place, and therefore, we simply removed the intimal flap in the dissection model to create a model of the undissected geometry. This was justified by the acute nature of the aortic dissection. In acute dissection, there is minimal remodelling of the lumina and septum. Although the false lumen can potentially enlarge acutely due to its smaller thickness (Masuda et al. 1992; Fisher et al. 1994; Erbel et al. 2001; Khan and Nair 2002), this expansion was not noted in this study. Second, we neglected the potential responses of both the baroreflex and the cerebral auto-regulation (Lau and Figueroa 2015), which are likely to react to changes in aortic pressure and upper branch flow following aortic dissection. Ignoring the baroreflex and local auto-regulations resulted in assuming identical inflow and outflow boundary conditions for both the dissected and undissected cases.

Since no flow data were available prior to the onset of the dissection, it was assumed that the cardiac output remained unchanged pre- and post-dissection. This assumption implies that the heart adapted to changes in afterload with the overall objective to maintain the cardiac output constant.

The heart model allows the estimation of stroke work, but as there was no image data available on ventricular volumes, a number of assumptions were made for the heart lumped-parameter model such as end diastolic volume and preload. Our heart parameter models were taken from a previously published study (Lau and Figueroa 2015). As such, the stroke work shown is an estimation of the cardiac workload. Future studies may benefit from the acquisition of cine image data of the heart.

## Conclusions

In this study, multi-modality image data were used to create and validate a detailed computational model of haemodynamics in the human dissected aorta. This is the first study in which numerical predictions are quantitatively compared against PC-MRI data in the descending aorta. Our results also show that secondary communicating tears, particularly larger tears, have a significant impact on haemodynamics in the descending and thoracic aorta.

There is a clear need for better imaging and computational methods to produce more accurate anatomical and physiological models of aortic dissection. Enhanced image-based simulation methods hold the key to improving our current understanding of the biomechanics of this complex disease. This better understanding will make it possible to devise better strategies for the long-term management of patients with chronic dissection, allowing for the identification of potential areas of aortic growth, ensuring visceral organs are adequately perfused and potentially aiding in surgical planning.
